# Enhancing Carbon Acid pK_a_ Prediction by Augmentation of Sparse Experimental Datasets with Accurate AIBL (QM) Derived Values

**DOI:** 10.3390/molecules26041048

**Published:** 2021-02-17

**Authors:** Jeffrey Plante, Beth A. Caine, Paul L. A. Popelier

**Affiliations:** 1 Lhasa Limited, Granary Wharf House, 2 Canal Wharf, Leeds LS11 5PS, UK; jeffrey.plante@lhasalimited.org; 2 Manchester Institute of Biotechnology (MIB), 131 Princess Street, Manchester M1 7DN, UK; bethan.caine@benevolent.ai; 3 Department of Chemistry, University of Manchester, Oxford Road, Manchester M13 9PL, UK

**Keywords:** pKa prediction, ab initio, bond length, carbon acid

## Abstract

The prediction of the aqueous pK_a_ of carbon acids by Quantitative Structure Property Relationship or cheminformatics-based methods is a rather arduous problem. Primarily, there are insufficient high-quality experimental data points measured in homogeneous conditions to allow for a good global model to be generated. In our computationally efficient pK_a_ prediction method, we generate an atom-type feature vector, called a distance spectrum, from the assigned ionisation atom, and learn coefficients for those atom-types that show the impact each atom-type has on the pK_a_ of the ionisable centre. In the current work, we augment our dataset with pK_a_ values from a series of high performing local models derived from the Ab Initio Bond Lengths method (AIBL). We find that, in distilling the knowledge available from multiple models into one general model, the prediction error for an external test set is reduced compared to that using literature experimental data alone.

## 1. Introduction

The fast calculation of complex molecular properties has been a goal of chemists for some time. Some of the first examples are found in the seminal papers of Hansch and co-workers [1,2]. Since then, many different techniques have been applied to a multitude of problems in chemistry from predicting the log P of a compound [3] to using complicated 3D descriptors to predict hERG activity [4]. In essence they all follow a similar methodology: gather the data that are available for the target of interest, choose an approach to featurise the molecules and use a machine learning technique to map this molecular representation to the endpoint of interest. Finally, evaluation of the performance of the model is performed on an external data set. By the nature of relying on experimental data to train the computational model, the performance is best when query compounds are drawn from an area of chemical space similar to that of the training set, i.e., for compounds that do not fall outside of the models’ applicability domain. In order to build a more general model, more training data are needed from a diverse chemical space. If one has unfortunately exhausted the available data then more experiments need to be run in order to gather more data, requiring both significant time and expense. An alternative approach combines the knowledge across multiple models by training using data predicted from suitably high-performing models [5]. In the current work, we investigate the use of highly local, high-performing models, using quantum chemical descriptors calculated at the B3LYP/6-311G(d,p)/CPCM level of theory to train a faster, more general model. The goal is to enable prediction of carbon acid pK_a_ values with acceptable speed (<10 ms per compound) for a high throughput setting, with enhanced accuracy.

A plethora of different methods has been investigated to predict the acidity of small (<50 atoms) organic molecules. They range from exceedingly precise quantum mechanical calculations over multiple different poses of a molecule to more simple linear-free-energy methods, or to even simpler partial-least-squares methods using calculated descriptors [6]. Each of these different methods is associated with different computational times and accuracy. Liao and Nicklaus [7] have compared the accuracy of nine different commercial methods but the authors considered only a single example of a carbon acid in their test set.

Part of Lhasa Limited’s software portfolio involves the prediction of chemical degradation under forced conditions [8]. A number of transformations are initiated through the deprotonation of a carbon. For that purpose, we have developed patterns to locate carbons that would have a sufficiently low pK_a_ to allow for deprotonation. These patterns contain the usual suspects of a carbon next to a ketone, sulphone, nitrile and others. However, using a pattern is a blunt instrument without allowing for the fine gradient that could be found with knowledge of the actual pK_a_. Therefore, we aimed at developing a system where we can calculate an estimated pK_a_ for the carbon acids and then use that pK_a_ in our likelihood system to assign a score for the transformation. In that vein, we developed an atom-typed method that is of sufficient accuracy as well as speed, but we quickly exhausted all of the available pK_a_ data for carbon acids. Hence the model, while functional for our purpose, could not improve its performance without additional data, which are currently not available in the public domain. The pK_a_ data does exist, but it is held in private data silos as shown by the collaboration between Simulations Plus and Bayer where they were able to use the pK_a_ data at Bayer to build a well performing model [9]. The SAMPL6 [10] challenge recently completed, but none of their test compounds included a single carbon acid, and none of the methods described in that issue (Journal of Computer-Aided Molecular Design, Vol. 32, No. 10, October 2018) were trained with any carbon acids.

Our proposed method of overcoming the hindrance described above is to generate virtual pK_a_ data for compounds using a sufficiently precise prediction method. In order to do so, we calibrate a specific local model, which is trained on the information in a narrow range of chemical space, and we then use that model to generate calculations for virtual molecules that lie within the domain of the model. For such virtual molecules, which are chemically valid but for which no experimental data are currently known, validation of the accuracy of the predictions is only inferred implicitly, via a reduction in prediction errors for the general model on an external test set. If this approach is taken, then the predictions must perform with excellent accuracy because any errors in the calculated training data will result in compounded errors from the final learned model. This is not the first time that calculated data have been used to train a model, [5,11] but this is the first time that Ab Initio Bond Lengths (AIBL, pertaining to the use of bond lengths as descriptors), have been used in this context. This quantum-chemically derived methodology operates in a small area of chemical space to generate data for a congeneric series with diverse substituent groups. These hypothetical data are then fed into our distance spectrum-based regression model, which has a more general applicability domain. Thus, the goal of using the calculated data is two-fold: (i) increase the accuracy of the model, and (ii) increase the coverage of the model.

In any Quantitative Structure Property Relationship study the modeller must choose how to encode structural information before using a regression algorithm to map this description to a certain endpoint. In many applications the compound is represented as a series of binary digits representative of the 2D structure. To this end, Extended Connectivity Fingerprints, where the structure is represented by means of circular atom neighbourhoods encoded into a specific length bit vector, are a common choice. Such representations may allow for a performant general model to be constructed, but at the cost of more detailed information pertaining to variations in electronic effects of substituents on the propensity for dissociation. Such information may only be accurately captured using molecular representations derived from quantum chemical calculations.

Examples of featurisation using 3D structure occur frequently in the field of learning models that predict quantum chemical properties. This area of research aims for the fast prediction of properties that would usually require a long computational time to obtain using standard quantum mechanical methods [12,13,14]. For example, recently the Isayev group used modified Behler-Parrinello symmetry functions to encode single-atom atomic environment vectors. These atomic level feature embeddings were then used as input to neural networks to build a potential called ANI-1, which has been shown to perform as well as a DFT calculation [15,16]. Graph Neural Networks have also been applied to learn molecular potentials, with one recent example using directional message passing to embed information about distances and angles between atoms in molecules, and spherical Bessel functions and spherical harmonics to construct physically based molecular representations. The prediction of pK_a_ as an endpoint in a QSPR model has been approached using molecular descriptors of both two and three dimensions. In our previous work [17,18,19,20,21,22,23], we have demonstrated that small variations in QM-derived bond distances may be mapped linearly to pK_a_ values. This so-called Linear Free Energy Relationship may be explained by a variation in the electronic distribution in the common substructure of the series, as peripheral substituent groups are altered. We suggest that using interatomic distances as descriptors to predict pK_a_ variation provides a more detailed description of electronic differences between substructures of similar compounds, such that differences in the thermodynamic process of deprotonation can be predicted to a high degree of accuracy. Despite this high accuracy in this narrow region of space, many hundreds, if not thousands of local linear models would have to be constructed to provide reasonable coverage of chemical space to make this approach generally applicable. We exploit the highly accurate predictions of the AIBL approach to increase the accuracy and coverage of our faster and more generalisable regression model, whilst retaining the speed advantage in running a prediction.

The workflow for constructing these highly accurate linear models consists firstly of locating clusters of compounds that are structurally highly similar, with corresponding experimental pK_a_ information, and calculating low-lying conformations to determine statistically significant (according to Boltzmann distribution) bond lengths. Electronic structure calculations are carried out using Density Functional Theory (B3LYP/6-311G(d,p)), which requires a significant, but not excessive, computation time. Bond lengths obtained from low-lying geometries are then mapped to the corresponding pK_a_ values to construct highly correlated linear regression models using only a single bond length. The equation (of the form pK_a_ = m*R(X − Y) + c) describing this relationship may then be used to determine the pK_a_ of unknown compounds. This method has been applied to many different functional groups and has been shown to provide a prediction accuracy of +/−0.5 log units. The strength of AIBL lies in the ability to calculate highly precise bond lengths such that tiny deviations of bond distances within the common fragment correspond to analogous trends in acidity/basicity, with a well-defined coverage area for each model. This model is then applicable to predict pK_a_ values for similar compounds containing a core chemical feature.

As speed is of the essence, the Lhasa pK_a_ methodology uses an atom-typed regression model where each different type of atom has a defined effect on the pK_a_ of the atom undergoing a deprotonation event. A molecule is subdivided into its component atoms and by using the topological bond distance to the pK_a_ centre we can estimate the impact that each atom has on the pK_a_ of the molecule. The atom-typing protocol is described in detail in the Supplementary Materials but, briefly, the atom type encodes the atom as well as a small amount of the local environment to account for steric and electronic considerations, which are known to affect pK_a_. The coefficient for each atom-type is learned from a simple linear regression from the feature vector describing each deprotonation centre. In that manner each prediction simply generates the desired feature vector from the molecule and then applies the coefficients in turn to calculate the pK_a_ for the deprotonation of the desired carbon. This approach results in a prediction time that is on the order of milliseconds per compound making the pK_a_ prediction suitable for running in a batch mode on thousands of compounds.

## 2. Results and Discussion

Each experiment was designed to build on the outcome of the previous experiment. In other words, we investigate the performance improvement by the successive addition of virtual compounds, thereby increasing the size of the training set with each addition. The training statistics are provided in Table 1, which also includes the number of compounds considered “inDomain” in the test set. A molecule is considered “inDomain” if the distance spectrum for the ionisation site only contains atom-types for which a coefficient has been calculated. Otherwise, the coefficient is assumed to be zero; a prediction is then still made but it should be used with caution. The calculated compounds were separated across 3 different datasets, which represent the results of 3 different AIBL models: deprotonation of sulphone-carbonyls, nitrile-carbonyls, and cyclic diketones (respective SMILES strings: S(=O)(=O)C*C(=O), N#CC*C(=O) and C1(=O)C*C(=O)CCC1 where C* is the site of deprotonation).

After the sulphone-carbonyl model was established using the C–C bond lengths of 14 compounds, the first set of virtual compounds were constructed. This initial set of compounds incorporated multiple nitro- and multiple amino-aromatic moieties, to extrapolate outside of the range of the AIBL model to extreme pK_a_ values. This initial set also contained compounds that were more focused on the diversity of atom-types in order to increase the number of atom-types available in the model and widen the applicability domain. The second set of virtual compounds consisted of nitrile-carbonyl derivatives, chosen to extend the pK_a_ range and atom-type diversity. The third virtual set consisted of diverse compounds calculated from a previously prepared AIBL model of cyclohexanediones and cyclopentanedione derivatives [24].

Overall, the inclusion of all virtual compounds increased the number of atom-types used for the model from 49 to 60, while the size of the training set increased from 234 pK_a_ points to 416 pK_a_ points. Overall, the number of compounds considered “in the domain of the model” increased from 221 to 256, compared to 316 in the entire test set. A prediction is considered in domain if it only contains atom-types for which it was able to learn a coefficient. The R^2^, or coefficient of determination, of the solution, found via the QR decomposition, also increased slightly from 0.869 to 0.877. This increase shows that the additional atom-types make the model better capture the variance in pK_a_ from the training set. The increase is modest but significant because the QR decomposition algorithm is a deterministic calculation, hence one obtains the exact same solution from the same set of input data, each time the calculation is performed. The number of different atom-types found in the log P training set of Werner and Plante [5] was 181, which gives an estimated upper bound on the number of different atom-types that are likely to be found in pharmacological chemical space. Insufficient pK_a_ data exists in the public sphere to reach this number of atom-types for carbon acids, but with judicious selection of virtual compounds it is an achievable goal for the future. As more data are incorporated into the training set, the QR decomposition will account for more atom-types and find a better solution.

Table 2 gives the performance improvements, showing the root mean squared error (RMSE) for the test set across each successive addition. These errors are examined in terms of three factors: (i) the overall performance of the test set, (ii) the local performance improvements in the specific domains that are added, and (iii) the performance of molecules that fall outside the chemical space where AIBL has provided virtual compounds. Notably, the first addition (set 1), which consisted of the sulphone-carbonyl compounds, resulted in a significant improvement in prediction accuracy in that specific area of chemical space, reducing the RMSE from 3.43 to 1.49. Importantly, the improvement was not limited to that domain and instead was also observed for compounds that were not sulphone-carbonyls, as evidenced by the RMSE reducing from 3.05 to 2.78 for compounds that are not carbonyl-sulphones (Table 2). This is likely a result of the additional atom-types allowing for a more optimal solution to arise from the QR decomposition that is closer to the impact each atom-type would have on the pK_a_ centre. Despite this reduction in prediction errors, the overall performance of the model for all carbon acids is still far from ideal. One reason for this poor performance may be due to inconsistent experimental conditions (e.g., solvent, temperature) for values used to train. Unfortunately, this is an unavoidable state-of-affairs for predicting carbon acids until more experimental data become available.

Despite the overall performance being poor, it is encouraging to note that through the addition of AIBL-derived compounds to the training set, 22 more compounds in the test set are brought into the applicability domain. The addition of the nitrile dataset (set 2) further increased coverage by 15 compounds, but also decreased the performance slightly to a RMSE of 2.82 (coming from 2.62) for all compounds (and 1.75 for the nitriles). However, this new value is still below the 2.96 of the original training set. Simultaneously, the coverage has increased with the addition of set 2, but it is possible that the diversity of atom-types in the training set is still missing key areas of chemical space relevant to test compounds, resulting in a slight decrease in performance. Another possibility is that certain atom types are only found within this data addition and that the solved coefficients are possibly not truly reflective of the impact on the pK_a_. This would resolve if they were present in other deprotonation centres. When the final, 24 compound, di-carbonyl dataset (set 3) is added to the training set, we once again observe a subtle amelioration in performance, as reflected in the decrease in RMSE. This subtlety in the RMSE reduction suggests perhaps that the training set already has enough compounds to cover that area of chemical space, which is likely because the majority of the data consists of carbons that are alpha to at least one, but frequently two, carbonyl moieties. It is also important to note that the solution was found using a QR decomposition, which means that it is impossible to generate error bars on the RMSE values because the calculation is deterministic, resulting in exactly the same solution when the exact same training data is used.

To ensure that the model is valid and not a chance-correlation, Y-scrambling was performed. We randomly shuffled the pK_a_ values amongst the training set and relearned and validated the model 1000 times. This scrambled model performs with a RMSE of 11.44 ± 4.81 across all 1000 replicates, which shows that the model does not consist of a chance correlation. As a baseline method to compare against, we tried to learn a model using ECFP fingerprints generated from RDKit^24^ in Knime (knime.org). Using this combination, the model was able to predict the test set with a RMSE of 8.582, showing that the distance spectrum is good at capturing the required information to predict pK_a_. The poor performance is not surprising because there is no information on which atom is undergoing a deprotonation event, and instead, the ECFP fingerprints are just encoding information on the entire molecule. We then examined how well a Random Forest of 100 trees captures the information in the ECFP fingerprints and such a model performs much better with a RMSE of 2.842, nearing the performance of the Lhasa model. When using the distance spectrum with a Random Forest of 100 trees, the performance improves again to a RMSE of 2.67, beating the simpler linear models’ performance of 2.74 but not by enough to switch to the more complicated model.

**Figure 1:**
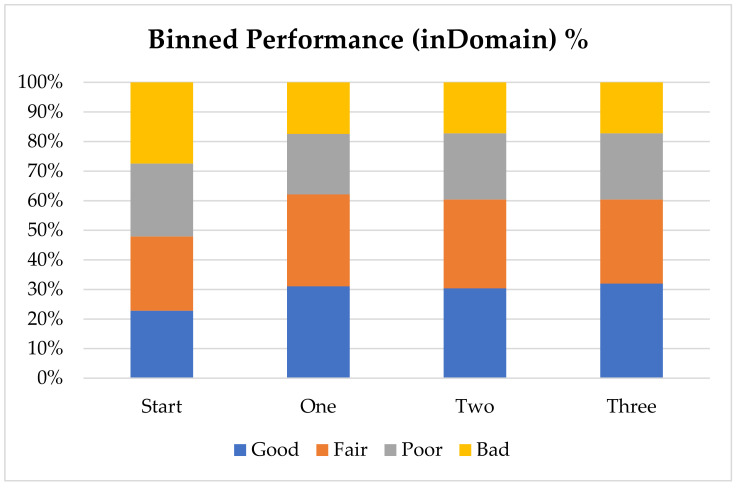

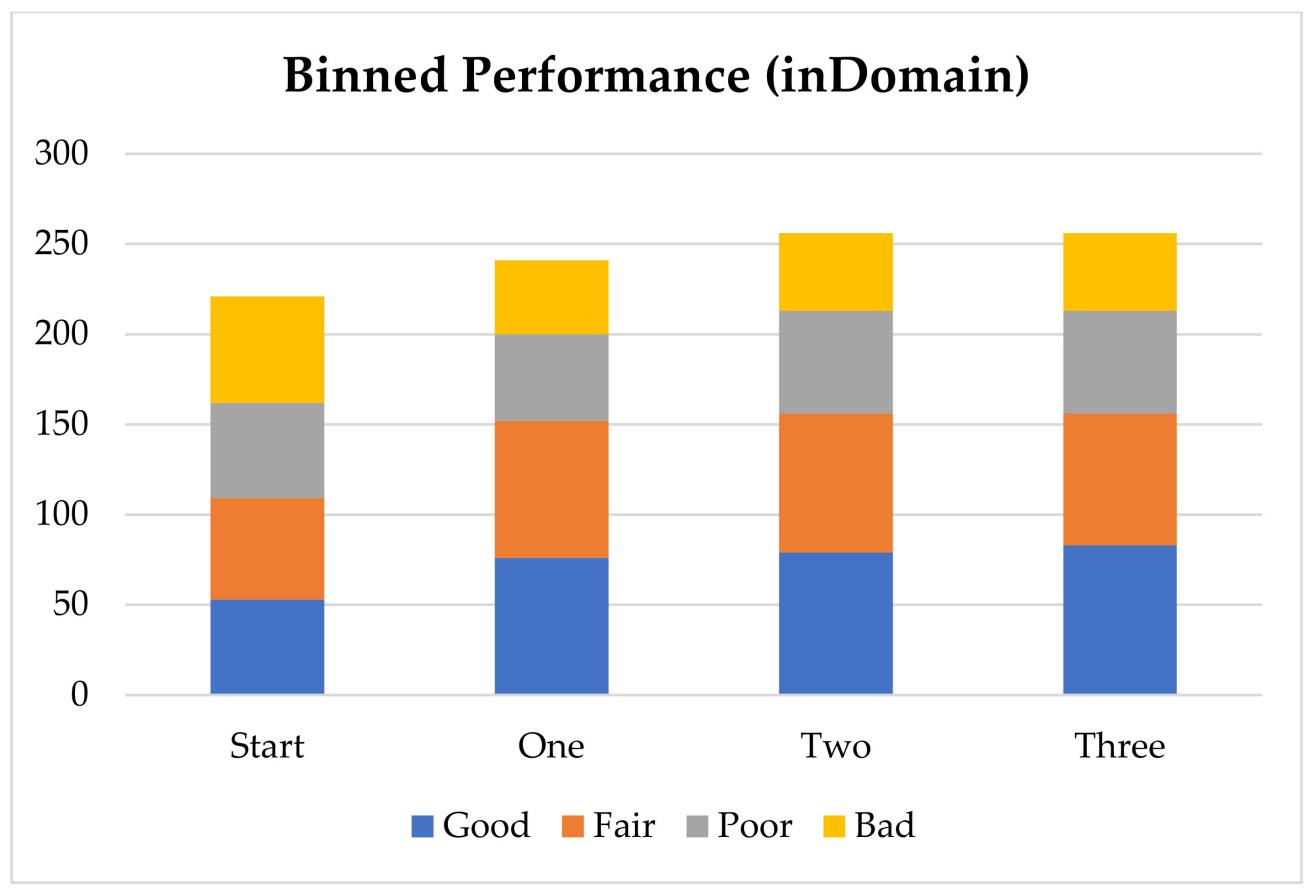
Binned performance stats by count (**top**) and by percentage (**bottom**) (if the absolute error is less than 1 pK_a_ unit then “Good”, 1 < Fair < 2, 2 < Poor < 3, Bad > = 3).

In order to further analyze the performance of the model with the addition of virtual compounds, we have binned the results by absolute error (Figure 1). In this case we consider a prediction “Good” when the absolute error is less than 1 pK_a_ unit, “Fair” when it is between 1 and 2 pK_a_ units, “Poor” when it is between 2 and 3 pK_a_ units and “Bad” when the absolute error is larger than 3 pK_a_ units. The final results show that for nearly 60% of the “inDomain” predictions the error is now less than 2 pK_a_ units. Furthermore, predictions classed as “Good”, consisting of those compounds with an absolute error of less than 1 pK_a_ unit and shown in blue in Figure 1, have increased with each additional dataset, while those with errors classed as “Bad” have steadily decreased. Given the trends we describe here, we expect that with a few more targeted AIBL models (for the sparsest regions of chemical space represented by the training set), the worst performing compounds will move into the better half. This will require careful consideration of the compounds being calculated as well as the expansion into new AIBL models hitherto undeveloped.

### Coefficients from QR Decomposition Solution

**Figure 2:**
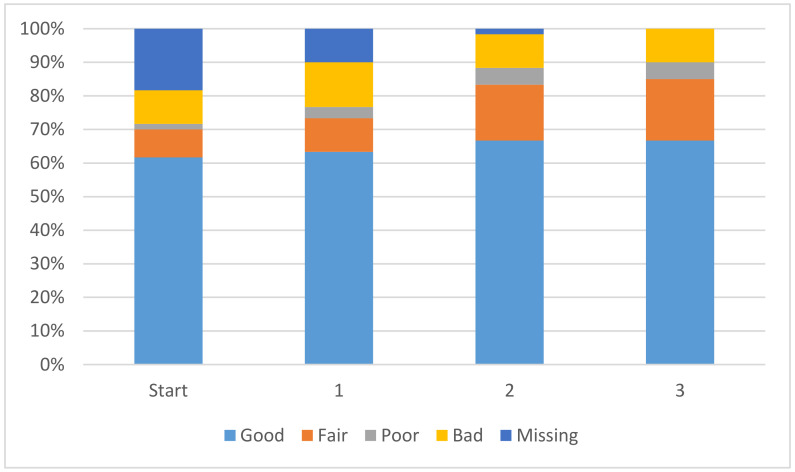
Percentage graph showing the reduction of “Bad” coefficients (yellow) (Light Blue < 10, Orange < 20, Grey < 30, Yellow > = 30, Dark Blue Missing coefficient).

Another beneficial outcome of the additional data is that the atom-type coefficients have improved significantly. Any linear model consists solely of these coefficients and they represent the impact that each atom-type has on the pK_a_ of the ionizing centre. It is desirable for each coefficient to have the smallest magnitude possible, while still allowing for accurate predictions, such that no single coefficient could have a major impact on the pK_a_ calculation. The improvement in coefficients is displayed in Figure 2. The overall magnitude of the coefficients is decreasing, leading to a solution where each coefficient will have a smaller and smaller impact on the overall pK_a_ value. In Figure 2 the coefficients with an absolute magnitude of less than 20 have increased overall and end up encompassing 85% of the total coefficients. Further discussion is available in the Supplementary Materials.

## 3. Methods

### 3.1. AIBL

Compounds of the carbon acids subset of the atom-type coefficient matrix model were represented as ECFP4 fingerprints and clustered using the Butina algorithm using RDKit [25]. The clusters were manually inspected to identify sets of congeneric series of a sufficiently large number containing a common site of dissociation. Three series were identified: sulphone-carbonyls, nitrile-ketones and cyclic diketones. The experimental data for these compounds were obtained from various literature sources and are referenced later. Next, an ensemble of 3D conformers was generated using RDKit. Each conformation was geometry-optimised at B3LYP/6-311G(d,p)/CPCM level using GAUSSIAN09 and the most stable geometry was identified by ranking total energies [26]. Bond distances around the protonation site of this geometry were then extracted and regressed onto experimental pK_a_ values. The linear regression equation of the bond length-pK_a_ model with the highest r^2^ value was then calculated, using only a single, selected bond length as the input feature.

### 3.2. Virtual Molecules

New compounds were then manually designed and constructed (conformers generated and subsequently geometry-optimised, following the procedure outlined above). The motivation for these virtual compounds was to add, to the common core of the congeneric series, substituent groups of novel character (i.e., differing to those already featured in the training set), with a wide variety of atom-types (atom numbers and local environments). In this sense, we expanded further the applicability domain of each model.

### 3.3. Sulphone-Carbonyl Model

**Figure 3:**
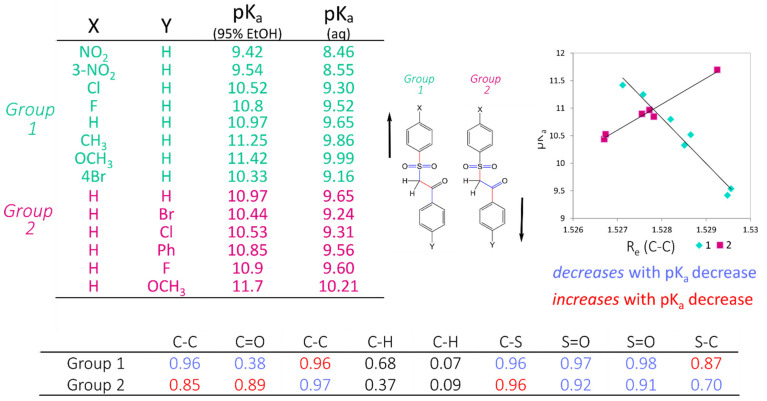
Investigations into the AIBL modelling of sulphone-carbonyl compounds showing the R^2^ correlation of the bond length to the pK_a_.

Fourteen datapoints were found for compounds that contained the sulphone-carbonyl moiety (SMILES string S(=O)(=O)C*C(=O) where C* is the site of ionisation) as shown in Figure 3. After the geometries were calculated they fell into two distinct groups: (i) those with a substituent on the aromatic ring on the sulphone portion (Group 1), and (ii) those with a substituent on the aromatic ring on the carbonyl portion (Group 2). The pK_a_ values for these compounds were given in 95% aqueous ethanol but were corrected to the water using the correlated linear relationship between experimental values obtained in water and aqueous ethanol, respectively. After calculating the precise geometries as described above, it was found that the C–C bond between the carbonyl and the site of ionisation had the greatest correlation (as demonstrated by the calculated r^2^ value) with the pK_a_ when split into two subsets, labelled Group 1 and Group 2 in Figure 3. Interestingly, two lines of best fit that emerge have gradients of opposite signs: a negative gradient for compounds substituted on the phenyl–SO_2_ terminus and a positive gradient for those with substituents at the phenyl-carbonyl terminus. The corresponding linear equations for the two lines-of-best-fit were used independently to generate virtual compounds for later inclusion into the distance spectrum model training set.

### 3.4. Nitrile-Ketone Model

**Figure 4:**
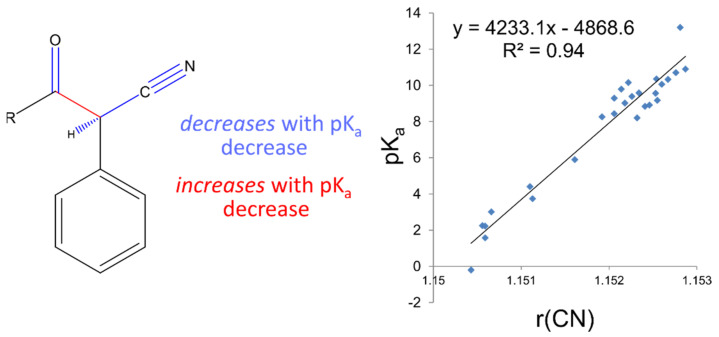
Modelling the nitrile-containing compounds.

Next, pK_a_ values were obtained for a series of carbon acids where the site of ionisation is adjacent to a nitrile group (SMILES String N#CC*C=O where C* is the site of ionisation). Figure 4 shows that a general linear model can be built that correlates the nitrile bond length with the pK_a_. The correlation for this model was not as high as has been obtained for previous models although we can surmise that it has a wider applicability due to the higher number of compounds used for the model and the larger range of response values (pK_a_). The full set was further subdivided into two groups depending on the character of the R group (Figure 4), R=H, OEt, OPh and Me, for which the correlation coefficient with pK_a_ was found to be almost unity (r^2^ = 0.98), but the interpolation space is more restricted by the reduced response range. These pK_a_ values were also present across aqueous DMSO mixtures and again a correlation was found, and the pK_a_ values were corrected. The best correlating bond was again found to be the nitrile bond, which contracts with decreasing pK_a_ (Figure 4). The resultant models were used to generate a further 97 compounds for inclusion into the training set across the two domains.

### 3.5. Cyclic Diketone Model

This model has been described previously [24] and 24 compounds were calculated for inclusion into the dataset and can be found in the complete training set included with the Supplementary Materials. The bond length identified as most performant was the C–O bond of the keto-enol tautomer in the anti-conformations, which had an r^2^ of 0.72 for r(C–O) vs pK_a_ for 49 training compounds, a 7-fold CV RMSEE of 0.57 and RMSEP for an external test set of 22 compounds of 0.24 log units.

### 3.6. Lhasa’s pK_a_ Method

**Figure 5:**
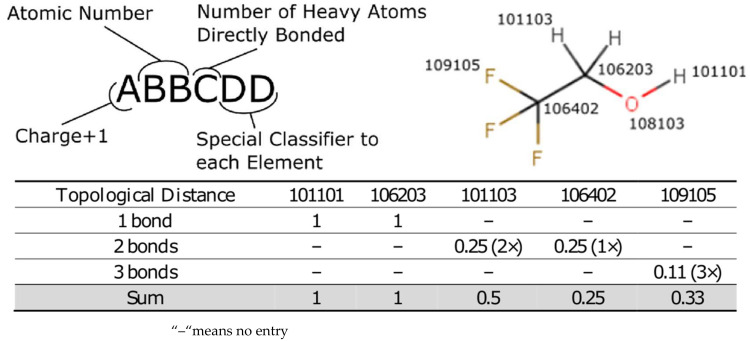
Atom-type description and an example of a distance spectrum calculation. This distance spectrum consists of the sum (one for each atom-type) of the inverse square of the topological distance to the pK_a_ centre, which is the oxygen (ionisation centre). Note that the oxygen (108103) is not included in the calculation of topological distances because it is obviously zero.

The Lhasa pK_a_ prediction method is an extension of the company’s log P prediction methodology [5], which uses at its core a system for generating different atom-types representing the local environment around each atom. Briefly, each atom is assigned a tag, which consists of a number in the format of ABBCDD. Figure 5 explains the meanings of A, BB, C and DD and shows an example of how this atom-typing works. Further details on the atom-typing scheme are summarised in the Supplementary Materials as well as in our log P paper.

The model is trained using data in the form of a pK_a_ value along with the atom involved in the ionisation event, which has been manually assigned. A distance spectrum is generated for the molecule from the assigned ionisation atom. This distance spectrum consists of the sum (one for each atom-type) of the inverse square of the topological distance to the pK_a_ centre (oxygen or 108103), as exemplified in Figure 5. Essentially, after each atom in the molecule has been atom-typed, the through-bond distance to the ionisation site is calculated. This integer value is inverted and squared to generate the fractional impact that the atom will have on the ionisation. These impacts are summed to generate a single feature vector consisting of the sum of all the distances to the ionisation centre by atom-type, highlighted in grey in Figure 5. It was theorised that this procedure will yield the impact that each atom-type has towards the pK_a_ of the ionisation site, in a similar manner to Xing’s molecular tree structured fingerprints [27].

In order to generate the model, many different distance spectra are collated into a large matrix and subjected to Partial Least Squares (PLS) via a QR decomposition, using the JAMA library [28] written in the language Java, to generate a coefficient for each atom-type. This coefficient is the numeric representation of the impact that the atom-type will have on the protonation or deprotonation site. The resultant model is simply these coefficients, along with the method to calculate the distance spectrum. Once these coefficients have been obtained, running a prediction is as simple as the summation over all atoms of the coefficient for the atom-type divided by the square of the topological distance, which results in the theoretical pK_a_ prediction (Equation (1)).

(1) $$pK_{a}=\;\underset{\begin{array}{c} All\;\\ Atoms \end{array}}{\sum }\frac{\alpha_{Atom-type}}{Topological\;Distance_{atom}^{2}}$$

where, according to the Lhasa pK_a_ prediction method, α is a coefficient for the atom-type found from QR decomposition and the Topological Distance is the through-bond distance to the atom undergoing a protonation or deprotonation. Note that the potential sites are located using simple rules, and that each class of deprotonation or protonation results in a model for that domain. These models are quite broad: for example, Oxyacid, Amine Acid, Carbon Acid and Sulphur Acid for deprotonations, and Alkylamine, Aromatic Amine and Imine for protonation. In essence the overall investigation boils down to the question if we can use AIBL to generate virtual molecules to feed into the Lhasa pK_a_ method, both to improve its coverage and its performance. We wish to combine the knowledge contained in multiple AIBL models into our more generally Lhasa model, using the virtual compounds as the substrate to transfer the knowledge.

### 3.7. Training Set

The training set for the Lhasa method was obtained by manually digitising the contents of the books that contain important pK_a_ data [29,30,31]. If during the collection multiple values were present at 25 °C then the average pK_a_ was taken. Furthermore, if no results were given at 25 °C then the temperature closest to 25 °C was used. The pK_a_ values were all obtained either in water, or in a water/solvent mixture, and used without correction. These minor variations typically limit the accuracy of the final model, but this decision was deemed unavoidable, given the restricted amount of data available. For each pK_a_ value, a site of ionisation was manually selected showing the atom where the deprotonation will occur.

### 3.8. Test Set

The test set consists solely of compounds collected from Reaxys^®^ [32] by gathering up all of the compounds with disassociation constant data. There are no computed molecules present in the test set as they are only used to facilitate the transfer of knowledge from the AIBL models to the Lhasa model. Frequently there are multiple different values for compounds so there was a need to automatically find the average values, accounting also for the possibility that there can be multiple pK_a_ values for a compound. Therefore, to simplify the problem where multiple values were present, they were added to a sorted list of increasing amplitude. This list of values was simplified into the accepted pK_a_ values by using a damped averaging approach elaborated via an example in the Supplementary Materials. This large set was then predicted using the Lhasa method, to determine the actual atom where the ionisation event was occurring, as the Lhasa prediction returns both a calculated pK_a_ value along with the atom number from the structure. This compound list was then trimmed to include only those compounds with one site, alongside compounds where the number of sites was equal to the number of experimental values. These experimental values were matched to the atomic sites by locating the smallest error between any experimental and calculated pK_a_ value, which assumes to be the correct atomic site for that experimental value. Then the process is repeated using the next smallest error until there were no more experimental points left to assign. This dataset was then subsampled to include only the carbon acids, and finally it was curated manually to remove some incorrect assignments, for example, a pK_a_ value of −0.5 for acetophenone, which is obviously the pK_a_ of the protonated carbonyl.

## 4. Conclusions

This investigation into the use of predicted data to train a simpler model has borne useful fruit. We used two different approaches for the prediction of pK_a_ and were able to combine them to improve coverage in the carbon acid area of chemical space. One approach is the AIBL model, which is very accurate but requires long computational times and has a very focused applicability domain. The other approach is the Lhasa model, which is widely applicable and computationally fast but requires significantly more training data than what is available to generate a good model. We were able to distil the knowledge present in three different AIBL models, consisting of sulphone-carbonyls, nitrile-carbonyls, and cyclic di-carbonyls, into the more general Lhasa model.

There is the potential to generate many additional data points, which will greatly improve the pK_a_ modelling available from our fast distance spectrum model by leveraging the knowledge contained in the more computationally expensive AIBL model. Speed is important as pK_a_ prediction is a necessary component in pharmacokinetics modelling, specifically the mole-fraction of a compound in the neutral state at pH 7.4 and 6.5 for calculating absorption rates and Caco-2 permeability [33].

Whilst the improvements in performance are more pronounced within the domain of the additional compounds, the impact of new compounds does bleed out into the entirety of chemical space, which directly follows from the improved predictions calculated with the additional data. The improvement in coverage and performance detailed in this manuscript has resulted in a calculator suitable to use in our Zeneth software for predicting chemical degradation, replacing complicated patterns to locate acidic hydrogens. Further work is underway to optimise the performance of the Lhasa pK_a_ calculator, which will be detailed in a further publication.

## Figures and Tables

**Table 1:** Results of the matrix solving via QR decomposition. R^2^ is the coefficient of determination, which captures the variance caught by the model.

Experiment	pK_a_ Points	Number of Atom-Types	R^2^	inDomain
Start	234	49	0.8698	215
1	276	54	0.8715	235
2	392	59	0.8762	250
3	416	60	0.8775	250

**Table 2:** RMSE values of the carbon acids in the test set trained with additional incorporated compounds.

	Overall		Sulphone-Carbonyl		Nitrile-Carbonyl		Di-Carbonyl		Others	
Addition	All	inDomain	All	inDomain	All	inDomain	All	inDomain	All	inDomain
Start	2.96	2.92	3.43	3.44	1.99	1.84	2.11	2.06	3.05	3.01
Sulphone (1)	2.62	2.53	1.49	1.53	1.64	1.64	2.03	1.99	2.78	2.77
Nitrile (2)	2.82	2.67	2.48	2.48	1.75	1.75	1.94	1.92	3.02	2.90
DiCarbonyl (3)	2.74	2.58	2.07	2.07	1.70	1.70	1.94	1.93	2.92	2.81

## Data Availability

Data available upon request from corresponding author.

## Supplementary Materials

The following are available online, Section S1: Damped Averaging, Section S2: Atom-Typer, Figure S1: The DD values for each element of the atom-typer, Section S3: QR Coefficient Improvement, Section S4: Atom-type Coefficients, Table S1: Coefficients from the solved model.
