# Supplementary material for: Enhancing Carbon Acid pKa Prediction by Augmentation of Sparse Experimental Datasets with Accurate AIBL (QM) Derived Values
Source: Molecules. 2021 Feb 17;26(4):1048. doi: 10.3390/molecules26041048 (PMC7922142; doi:10.3390/molecules26041048)
Supplement: Supplementary file 1 [file molecules-26-01048-s001.pdf]

molecules

MDPI

Supplementary Materials

Enhancing Carbon Acid pK_a Prediction by Augmentation of Sparse Experimental Datasets with Accurate AIBL (QM) Derived Values

Jeffrey Plante [1], Beth A. Caine [2] and Paul L. A. Popelier [2,3,*]

[1] Lhasa Limited, Granary Wharf House, 2 Canal Wharf, Leeds, LS11 5PS; jeffrey.plante@lhasalimited.org
[2] Manchester Institute of Biotechnology (MIB), 131 Princess Street, Manchester, M1 7DN; bethan.caine@benevolent.ai
[3] Department of Chemistry, University of Manchester, Oxford Road, Manchester, M13 9PL
* Correspondence: pla@manchester.ac.uk

1. Damped Averaging

For each compound, all of the pK_a values gathered from the literature were sorted from the lowest to the highest value; the first value was taken and added to a growing array. The next value was examined and compared to the average of the array. If it differed by more than 2 pK_a units, the average of the array was added to the list of reduced values and the new value formed the start of a new array. Otherwise, it was added to the array and the next value was compared. In this fashion the multiple different values were condensed into exemplar values. For example, the list of (0.1, 0.2, 0.3, 2.5, 2.6, 3.0) would be condensed down into (0.2, 2.7) thereby allowing for minor experimental disagreement and using the average values for training purposes. This method has the potential to merge different pK_a values if they lie within 2 units apart. However, this condition is unlikely except for the case of certain zwitteronic compounds, and in those cases, it is an acceptable compromise to assign one pK_a value to the entire molecule to learn from.

2. Atom-Typer

The atom-typer generates an integer for each atom that encodes four pieces of information:

A. The formal charge of the atom.
B. The atomic number of the atom.
C. The number of non-hydrogen atoms covalently bound to the atom (i.e. a simple measure of sterics).
D. Information about the hybridization and local connectivity.

Overall, the integer takes the form of ABBCDD where:
A = formal charge + 1.
BB = atomic number (e.g. 01 = hydrogen; 06 = carbon; 09 = fluorine)
C = Integer count of non-hydrogen atoms covalently bound to the atom.
DD = Hybridization value that is dependent on the element being typed.

The formal charge portion is the charge +1 because the formal charge is unlikely to fall out of the range of ±1, so this just keeps the numbers positive. The formal charge, atomic number and non-hydrogen bonding count are essentially self-explanatory but the hybridization value requires further elaboration.

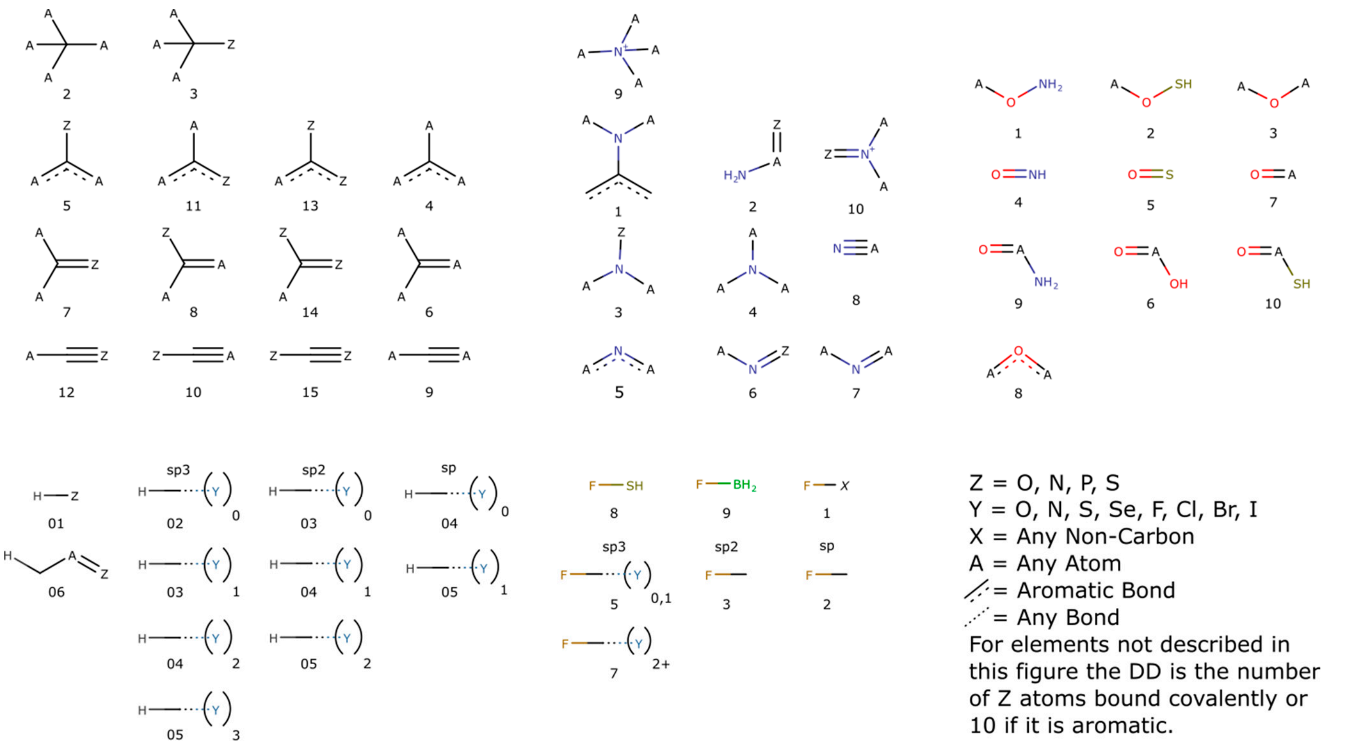

Figure S1. The DD values for each element of the atom-typer.

Each of the commonly observed elements in organic chemistry space has its own set of hybridization values. These are an attempt to classify the local environment of the atoms and thereby consider various electronic effects. For example, if a carbon is a sp^3 carbon then there are 2 types: 02 and 03 depending on if there is a highly polar group (Z = O, N, P or S) attached to it or not. For the sp^2 carbon types there are two major groups: aromatic and non-aromatic. Both groups are further subdivided into 4 specific bonding patterns with strongly polar atoms. In the case of nitrogen matters are similar, with a major addition being that an aniline-like moiety (01) and an amide like moiety (02) each also receive their own DD value, in order to reflect the specific electronic states of those two atom-types. Oxygens are separated out to split carbonyl types if they are part of amides, carboxylic acids, or thio-acids as each of those carbonyl groups exerts a different effect on a molecule. Fluorine has special cases as well, mostly to account for the case of CF_3 groups exerting a non-linear effect with the elimination of each fluorine. If an element is not present in Figure S1, then its default hybridization value is simply the sum of polar bonds to this element, with an additional 10 if the atom is considered aromatic.

3. QR Coefficient Improvement

When the data are limited, the possibility arises that a solution will be found where two coefficients are very large and with opposite signs. This occurs when two atom-types are always present together and not ever encountered individually in one molecule. An example of this situation are the two oxygen atoms present in the same nitro group, where one oxygen has a charge and the other does not, leading to their atom-type codes being 008104 and 108104. From Table S1 it is evident that a sub-optimal solution has been found for the initial model (Start). If one atom-type of the pair is encountered alone, as is the case for a N-oxide moiety, then a spurious value is generated (of the order of 1014) for the pK_a. This results in a nonsense prediction for the pK_a value. This pairing of coefficients is not a problem if the atom-types are always encountered together. However, they represent an undesirable solution if an atom-type is not present with its pair partner because an obviously incorrect prediction can occur. Examining the coefficients, excluding the coefficients for the nitro oxygens shows that the standard deviation of the remaining coefficients

decreases from 91.8 to 11.0 after the addition of virtual data. So, the QR decomposition is locating a more stable solution, without having large antagonistic coefficients. This means that the predictions are going to be more robust because, instead of having to encounter pairs of coefficients in concert to generate a good prediction, each coefficient reflects its own impact on the pK_a of the molecule better.

There are mathematical approaches to guide the Lhasa methodology towards finding the optimal solution, one of which was used with our log P work (reference 5 in main document). In that case every atom-type had 0.01 added to its occurrence in the matrix, resulting in a sparse matrix with a background value of 0.01. This modification in turn resulted in an improved solution for the log P work and is applicable as the rest of the entries were integers with a minimum value of 1. However, this approach is unsuitable for the current pK_a work because the values present in the distance spectrum range from 1 to 1/64 (0.015625) per atom. Hence, an addition of 0.01 represents a significant change in the values present, as opposed to the log P work where the minimum value present would be 1 such that 0.01 represents only 1% of the smallest value. Therefore, the only way out of this conundrum is to obtain more data, especially for certain atom-types, which would eliminate the previously described pairs.

4. Atom-type Coefficients

Table S1. Coefficients from the solved model.

Code	Start	1[a]	2	3
101101	22.25	24.96	28.58	29.43
101102	1.47	1.40	1.28	1.24
101103	4.00	4.21	4.18	4.18
101104	0.45	0.52	0.51	0.93
101105	3.35	3.18	3.18	3.14
101106	2.65	4.68	3.58	3.30
106102	1.89	2.07	2.12	2.09
106202	1.08	1.24	1.28	1.32
106302	1.91	2.12	2.14	2.12
106402	−3.67	−3.35	−3.38	−3.40
106103	−2.88	−4.25	−3.88	−3.33
106203	0.09	−0.16	−0.21	−0.26
106303	1.68	0.75	0.92	0.78
106403	−1.09	−4.00	−3.84	−3.18
106204	5.34	3.49	3.52	3.00
106304	−0.88	0.41	0.45	0.61
106305	−0.12	−3.26	−3.27	−2.91
106206	2.67	2.59	2.64	2.67
106207	3.75	3.96	4.10	3.85
106307	2.54	3.59	3.73	3.42
106211	−6.39	−1.34	2.77	1.99
106311	1.10	1.25	0.49	0.50
106212	9.53	9.72	9.86	9.48
107101	–	−44.06	−50.07	−51.21
107301	–	3.80	4.98	4.90
107102	−11.93	−14.83	−17.30	−17.59
107202	−2.80	−3.36	−4.29	−4.46
107302	3.43	4.19	4.97	5.42
107104	−7.24	−8.62	−10.45	−10.89
107304	−1.48	−1.83	2.51	2.65

107205	9.66	4.22	1.18	1.88
107206	–	324.92	117.74	147.30
107207	−7.11	−14.52	−19.68	−19.73
107108	−34.52	−35.05	−35.06	−33.81
207310	6.35	3.72	3.97	4.60
108102	438.42	71.78	−33.11	−31.87
108103	−13.09	−11.10	−12.84	−12.70
108203	7.09	10.04	10.04	10.92
108104	$5.40x10^{14}$	−469.71	−187.17	−231.39
008104	-5.40 $x10^{14}$	448.05	164.45	206.01
108105	−392.46	−90.17	−10.66	−11.38
108106	−12.70	−18.70	−19.15	−18.82
108107	−13.26	−16.92	−17.38	−16.12
108208	–	–	−0.89	0.59
108109	−19.13	−22.39	−23.68	−22.89
108110	–	–	−26.05	−21.69
109100	4.04	4.03	4.05	4.06
109102	–	−4.01	−4.73	−4.42
115300	–	–	10.41	10.31
115401	–	–	–	11.71
116100	–	–	−13.51	−14.44
116200	–	–	9.66	6.89
116401	197.95	46.48	6.79	7.10
116210	–	−8.57	−5.64	−5.20
117100	2.97	3.08	3.11	3.13
117102	−0.97	−6.64	−5.20	−3.79
134200	−3.26	−0.73	−0.37	0.16
135100	2.22	2.23	2.29	2.29
135102	−1.18	−8.15	−6.22	−1.81

[a] Table 1 of the main text provides the details of experiments “1”, “2” and “3”.
